# Rapidly reversible persistent long-term potentiation inhibition by patient-derived brain tau and amyloid ß proteins

**DOI:** 10.1098/rstb.2023.0234

**Published:** 2024-07-29

**Authors:** Tomas Ondrejcak, Igor Klyubin, Neng-Wei Hu, Yin Yang, Qiancheng Zhang, Brian J. Rodriguez, Michael J. Rowan

**Affiliations:** ^1^ Department of Pharmacology and Therapeutics, School of Medicine, and Institute of Neuroscience, Trinity College, Dublin 2, Republic of Ireland; ^2^ Department of Physiology and Neurobiology, School of Basic Medical Sciences, Zhengzhou University, 100 Science Avenue, Zhengzhou 450001, People's Republic of China; ^3^ School of Physics and Conway Institute of Biomolecular and Biomedical Research, University College Dublin, Dublin 4, Republic of Ireland

**Keywords:** Alzheimer’s disease, Pick’s disease, tau oligomers, amyloid ß protein oligomers, brain aqueous extract, long-term potentiation

## Abstract

How the two pathognomonic proteins of Alzheimer’s disease (AD); amyloid ß (Aß) and tau, cause synaptic failure remains enigmatic. Certain synthetic and recombinant forms of these proteins are known to act concurrently to acutely inhibit long-term potentiation (LTP). Here, we examined the effect of early amyloidosis on the acute disruptive action of synaptotoxic tau prepared from recombinant protein and tau in patient-derived aqueous brain extracts. We also explored the persistence of the inhibition of LTP by different synaptotoxic tau preparations. A single intracerebral injection of aggregates of recombinant human tau that had been prepared by either sonication of fibrils (SτAs) or disulfide bond formation (oTau) rapidly and persistently inhibited LTP in rat hippocampus. The threshold for the acute inhibitory effect of oTau was lowered in amyloid precursor protein (APP)-transgenic rats. A single injection of synaptotoxic tau-containing AD or Pick’s disease brain extracts also inhibited LTP, for over two weeks. Remarkably, the persistent disruption of synaptic plasticity by patient-derived brain tau was rapidly reversed by a single intracerebral injection of different anti-tau monoclonal antibodies, including one directed to a specific human tau amino acid sequence. We conclude that patient-derived LTP-disrupting tau species persist in the brain for weeks, maintaining their neuroactivity often in concert with Aß.

This article is part of a discussion meeting issue ‘Long-term potentiation: 50 years on’.

## 1. Introduction

Alzheimer’s disease (AD) brain is characterized neuropathologically by the deposition of relatively insoluble fibrillar aggregates of two proteins, amyloid ß protein (Aß) in largely extracellular plaques and microtubule-associated tau protein in largely intracellular neurofibrillary tangles. The amyloid cascade hypothesis posits a primary role for Aß in triggering subsequent tau pathology, synaptic loss and neurodegeneration [1,2]. Research on the amyloid-cascade hypothesis, including studies of the synaptotoxicity of more water-soluble non-fibrillar aggregates of Aß (Aß oligomers, oAß) [1,2], has played a significant role in the recent paradigm-shifting successful development of the first disease-modifying immunotherapeutics, lecanemab [3] and donanemab [4]. Although we await their complete real-world evaluation, it appears that this strategy will need to be augmented to achieve meaningful benefits for the majority of AD patients. Apart from enhancing their efficacy and safety profile, other complementary strategies need our urgent attention. One such approach is to target tau [5] especially in the context of its synaptotoxic interactions with Aß [6].

Pathological tau is very heterogeneous with many forms prone to aggregate into increasingly water-insoluble assemblies [7,8]. Preparations of recombinant human tau have been used to mimic some of these assemblies and certain tau aggregates potently inhibit long-term potentiation (LTP) in a manner similar to tau extracted from AD brain [9–11].

Although the synaptic plasticity disrupting action of the vast majority of minimally manipulated aqueous extracts of AD brain are known to be caused by oAß [12] (but see [13]), we recently found extracts from some cases of AD where tau, either alone [11] or in concert with oAß [14], was a significant synaptotoxin. In the latter cases, tau and Aß acted concomitantly lowering each other’s threshold for acute and persistent LTP inhibition. Because pathological tau, mainly an intracellular protein, appears to spread readily intercellularly in the brain [15–19], studying the *in vivo* synaptotoxicity of tau in such extracts is considered to provide a potentially powerful means of elucidating disease pathophysiology, complementing other approaches (e.g. [20,21]).

Here, we further explored the synaptic plasticity disrupting actions of soluble synaptotoxic full-length tau aggregates prepared from recombinant protein, after either sonication of pre-formed fibrils (sonicated tau aggregates, SτAs) [11,22] or disulfide bond formation (oligomer-enriched tau, oTau) [9], and that present in aqueous extracts of neuropathological brain samples, including AD and the tauopathy of Pick’s disease (PiD) [23,24]. We found that the threshold for acute LTP inhibition at CA3-to-CA1 synapses by oTau was lowered in APP-transgenic rats with elevated endogenous oAß [25]. Both SτAs and synaptotoxic tau-containing brain extracts caused a very prolonged disruption of plasticity after a single intracerebral injection. Remarkably, anti-human tau antibody rapidly reversed the persistent inhibition of LTP.

## 2. Results

### (a) Oligomer-enriched recombinant tau potently inhibits 400 Hz strong conditioning stimulation (sHFS-400 Hz) -induced long-term potentiation in amyloid precursor protein-transgenic rats *in vivo*

**Figure 1.**
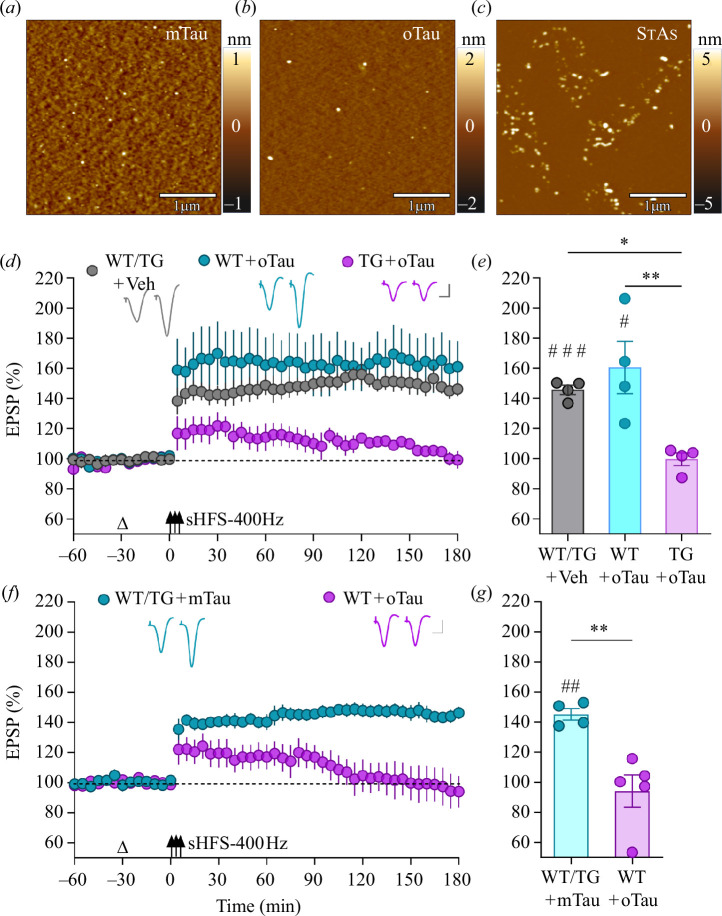
Acute inhibition of 400 Hz strong conditioning stimulation (sHFS-400 Hz)-induced LTP at hippocampal CA3-to-CA1 synapses by oligomer-enriched tau preparation (oTau) is enhanced in APP transgenic rats. (*a–c*) AFM height images of (*a*) tau monomer (mTau), (*b*) oTau and (*c*) soluble sonicated tau fibril-derived aggregates (SτAs), (horizontal scale bar = 1 μm; vertical height scales are 1, 2 and 5 nm in (*a*), (*b*) and (*c*), respectively). (*d–g*) *In vivo* electrophysiology, under urethane anaesthesia, in 5–7-month-old McGill-R-Thy1-APP transgenic (TG) and age-matched wild-type (WT) rats. (*d*,*e*) Acute intracerebroventricular (i.c.v.) injection (open triangle) of 4.9 pmol oTau inhibited strong conditioning stimulation (3 × 400 Hz trains, sHFS-400Hz; three arrows)-induced LTP in the TG rats (TG+oTau). By contrast, the LTP magnitude in oTau-injected WT rats (WT+oTau) was similar to the vehicle-injected animals (WT/TG+Veh). sHFS-400 Hz induces similar magnitude LTP in WT and TG rats. (*f*,*g*) A higher dose of 12.3 pmol oTau inhibited LTP in WT animals (WT+oTau), whereas sHFS-400 Hz triggered robust LTP in WT and TG rats injected with this dose of mTau (WT/TG+mTau). Summary bar charts in (*e* and *g*) show the magnitude of LTP during the last 10 min for time-course data in (*d*) and (*f*), respectively. Representative excitatory postsynaptic potentials (EPSP) traces at 10 min prior and 180 min post-sHFS-400 Hz. Scale bars: vertical, 1 mV; horizontal, 10 ms. Values are mean ± s.e.m., *^#^p* < 0.05, ^##^ *p* < 0.01, ^###^ *p* < 0.001 compared with pre-sHFS-400 Hz, paired *t*-test; *p < 0.05, **p < 0.01, one-way ANOVA followed by multiple-comparison tests (see also the electronic supplementary material, table S1 for full statistical information).

Tau aggregates prepared from recombinant full-length tau provide the greatest coverage of the many different lengths of endogenous tau found in AD brains. In the following sets of experiments, we tested the LTP disruptive actions of two different preparations of synaptotoxic recombinant full-length tau aggregates: (i) oTau prepared directly from tau monomers (mTau) and (ii) SτAs prepared from pre-formed tau fibrils [11,22]. Both forms of tau aggregates are known potent inhibitors of LTP [9,11]. As shown in figure 1*a*–c, tau monomers, tau oligomers and SτAs, deposited on mica substrates, were characterized by atomic force microscopy (AFM), as described in §4e. In figure 1*a*, tau monomers are discernible as discrete particles. The relatively low particle height makes it the least distinguishable from the background when compared with tau oligomers and SτAs, consistent with their monomeric state. Figure 1*b* presents particles having a greater height compared with the mTau sample (please note the increased height data scale). In figure 1*c*, the SτAs are clearly visible against the background with a more diverse range of particle sizes and shapes, including particles similar in appearance to oligomers, consistent with the use of sonication during their preparation from tau fibrils.

Knowing that synthetic oAß lowers the threshold for synaptotoxic tau to inhibit our standard high-frequency conditioning stimulation (HFS-200 Hz)-induced LTP [26], we wanted to determine if endogenous oAß also facilitates the inhibition of LTP by synaptotoxic tau. Here, we compared the effect of intracerebroventricular (i.c.v.) injection of oTau in wild-type (WT) and McGill-R-Thy1-APP transgenic (TG) rats. These TG rats provide a very complete model of AD amyloidosis [25] and have an age-dependent deficit in HFS-200 Hz-induced LTP mediated by oAßs, prior to fibrillar Aß deposition in plaques [27,28]. In order to assess the ability of oTau to affect LTP at CA3-to-CA1 hippocampal synapses in these less than 10-month-old APP-overexpressing animals before extensive plaque accumulation, we therefore used a relatively strong high-frequency conditioning protocol, consisting of three sets of trains of high-intensity stimulation at 400 Hz (sHFS-400 Hz), to trigger LTP that is not significantly impaired in TG rats compared with age-matched WT littermates [27].

Previously, we reported that SτAs are potent inhibitors of LTP induced either by 200 Hz-HFS or 400 Hz-sHFS in WT rats, with a threshold dose in the region of 1.2–1.4 pmol [11]. Consistent with previous reports of the LTP disruptive action of oTau *in vitro*, in WT rats injected 30 min previously with oTau (4.9 pmol, i.c.v.) the application of 200 Hz-HFS induced a decremental LTP (107 ± 1.3% at 3 h post-HFS; *p* = 0.0554 compared with pre-HFS baseline, paired *t*-test; *n* = 4; data not shown). Somewhat surprisingly therefore, the application of 400 Hz-sHFS after this dose of oTau in WT rats (WT+oTau, 4.9 pmol, i.c.v.) induced robust LTP (160.5 ± 17.4% versus 145.7 ± 3.2% elicited in vehicle-injected WT/TG+Veh control rats; figure 1*d,e*; see also the electronic supplementary material, table S1, for full statistical information). By contrast, in 5–7-month-old TG rats injected with oTau, the same sHFS protocol only triggered a transient potentiation (TG+oTau, 4.9 pmol, i.c.v., 99.6 ± 4.2%). Of note, in WT rats increasing the dose of oTau to 12.3 pmol strongly inhibited sHFS-induced LTP (94.2 ± 10.7% versus 145.1 ± 3.9% in rats treated with the same dose of mTau; figure 1*f,g*). This dose of oTau (12.3 pmol, i.c.v.) did not significantly affect baseline synaptic transmission (see the electronic supplementary material, figure S1). The apparent lack of effect of mTau on sHFS-induced LTP is consistent with our previous report that i.c.v. injection of relatively large amounts (16–19 pmol) of mTau failed to affect LTP induced by 200 Hz-HFS [11].

### (b) Effects of synaptotoxic tau-containing Alzheimer’s disease brain aqueous extracts on sHFS-400 Hz-induced long-term potentiation in wild-type and amyloid precursor protein-transgenic rats

**Figure 2.**
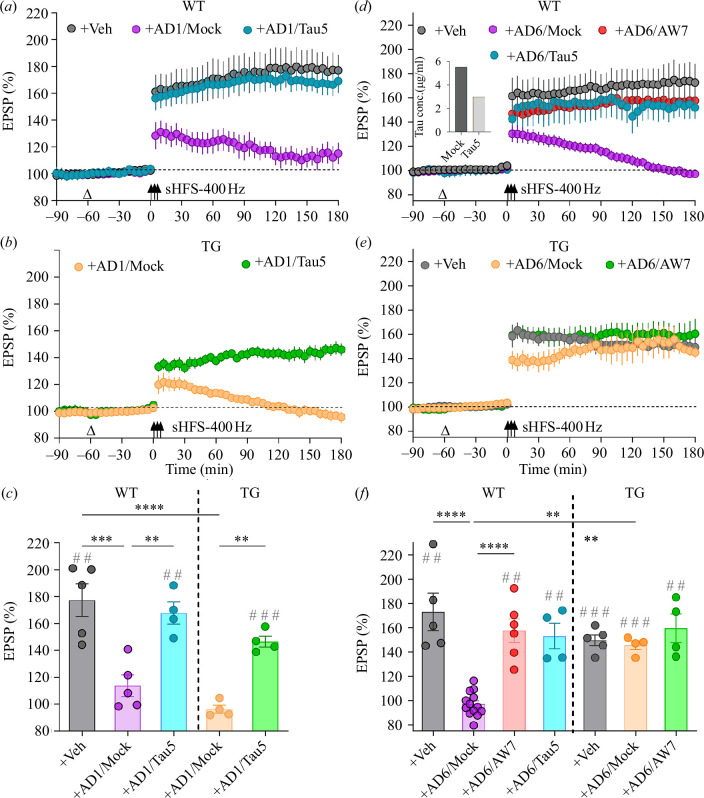
Acute effects of AD brain aqueous extracts containing synaptotoxic tau ± Aß on strong HFS-400 Hz-induced LTP in APP transgenic rats. Mock-immunodepleted AD1 extract (AD1/Mock), previously found to inhibit 200 Hz HFS-induced LTP in a tau-dependent, but Aß-independent, manner [26], inhibited strong conditioning stimulation (3 × 400 Hz trains, sHFS-400 Hz; three arrows)-induced LTP in both McGill-R-Thy1-APP transgenic (TG+AD1/Mock) and age-matched wild-type (WT+AD1/Mock) animals (*a–c*). sHFS-400 Hz induces similar magnitude LTP in WT and TG rats. On the other hand, AD6 extract, previously found to inhibit 200 Hz HFS-induced LTP in a tau- and Aß-dependent manner [14], inhibited sHFS-400 Hz-induced LTP only in wild-type (WT+AD6/Mock) but not in APP TG (TG+AD6/Mock) rats (*d–f*). Vehicle-injected animals (+Veh) served as interleaved controls. To address the involvement of tau and Aß in the inhibition of sHFS-400 Hz-induced LTP, AD brain extracts were immunodepleted with either the anti-tau monoclonal antibody Tau5 (+AD/Tau5) or with the polyclonal anti-Aß immunoserum AW7 (+AD/AW7). Inset in (*d*) shows tau concentration after immunodepletion. Values are mean ± s.e.m. ^##^ *p* < 0.01, ^###^ *p* < 0.001 compared with pre-sHFS-400 Hz, paired *t*‐test; **p < 0.01, ***p < 0.001, ****p < 0.0001 one-way ANOVA followed by multiple-comparison tests.

Next, we investigated whether synaptotoxic tau-containing AD brain affects sHFS-induced LTP in WT and TG rats. Because tau in brain aqueous extracts from different cases of AD inhibits 200 Hz-HFS-induced LTP in WT rats either in an Aß-independent or an Aß-dependent [14] manner, we tested examples of each. Extract AD1 acts in a purely tau-dependent manner ([11]; see the electronic supplementary material, table S2). In the case of mock-immunodepleted AD1 extract (AD1/Mock), when injected (10 μl, i.c.v.) 60 min prior to 400 Hz-sHFS, as shown in figure 2*a*–*c*, LTP was inhibited in both WT and TG animals (WT+AD1/Mock, 113.7 ± 8.0% and TG+AD1/Mock, 96.2 ± 2.9%). Importantly, removal of tau by immunodepletion (ID) with a mid-region-directed anti-tau monoclonal antibody (mAb), Tau5, prevented the inhibition of sHFS-induced LTP in both WT and TG rats (WT+AD1/Tau5, 167.8 ± 8.1% and TG+AD1/Tau5, 146.6 ± 4.1%), consistent with our previous findings for AD1 extract-mediated inhibition of HFS-200 Hz-induced LTP in WT animals.

By contrast, AD6 inhibits HFS-200 Hz-induced LTP in a manner requiring both tau and Aß ([14]; see the electronic supplementary material, table S2). Intriguingly and somewhat surprisingly, in the case of this extract, although an i.c.v. injection of 20 μl was sufficient to inhibit sHFS-400 Hz-induced LTP in WT rats (WT+AD6/Mock, 97.1 ± 2.8%), sHFS-induced robust LTP in TG animals injected with the same amount of AD6 extract (TG+AD6/Mock, 145.6 ± 3.6%; see figure 2*d*–*f*). In accordance with our previous findings, ID of either Aß with AW7 or tau with Tau5 prevented the inhibition of sHFS-induced LTP in WT animals (WT+AD6/AW7, 157.7 ± 9.6% and +AD6/Tau5, 153.3 ± 10.5%; see figure 2*d,f*).

### (c) Sonicated tau aggregate-mediated inhibition of long-term potentiation persists in both wild-type and amyloid precursor protein-transgenic rats

**Figure 3.**
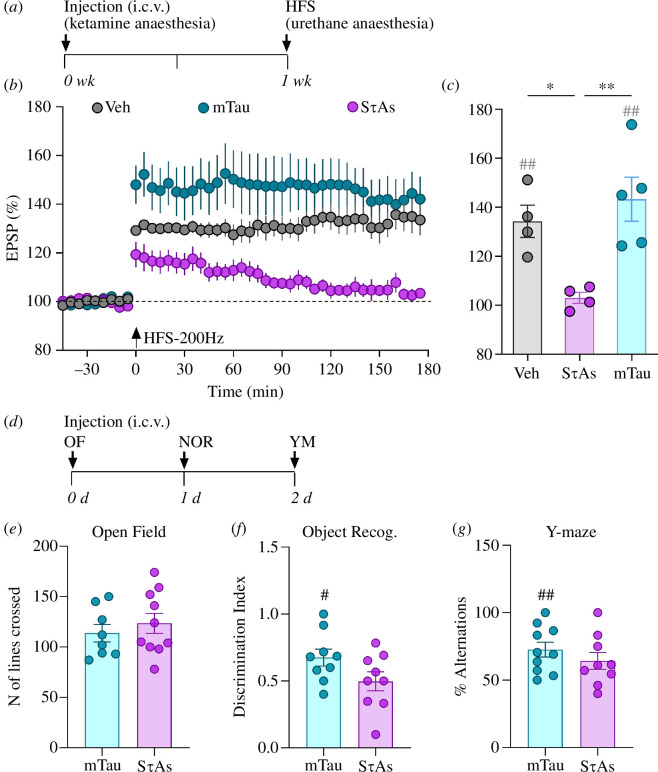
SτAs persistently inhibit standard 200 Hz conditioning stimulation (HFS-200 Hz)-induced LTP and disrupt cognition. (*a*) Study design. Seven days before electrophysiological recording WT animals received a single i.c.v. injection of vehicle or 19.6 pmol of either mTau or SτAs under recovery anaesthesia (ketamine/medetomidine). (*b*,*c*) Application of our standard HFS (single train at 200 Hz, arrow), under urethane anaesthesia, triggered stable LTP in vehicle and mTau-injected rats, whereas LTP was strongly inhibited by SτAs. Values are mean ± s.e.m. ^##^ *p* < 0.01, compared with pre-HFS, paired *t*‐test; *p < 0.05, **p < 0.01, one-way ANOVA followed by multiple-comparison tests. (*d*) Study design. WT rats received single i.c.v. injection of 19.6 pmol of either mTau or SτAs. The performance of rats was tested sequentially in an open field (OF) test, a novel object recognition task (NOR) and a Y-maze task (YM). Summary of (*e*) lines crossed in the OF, (*f*) discrimination index in the NOR and (*g*) alternations in the Y-maze. Unlike mTau-injected animals, the SτA-treated rats failed to show a significant preference for the novel object in the NOR recall test or alternation bias in the Y-maze. The # symbol stands for a statistical comparison with the hypothetical no-bias value (one-sample *t*-test). ^#^ *p* < 0.05; ^##^ *p* < 0.01. Values are mean ± s.e.m.

To investigate if the preparations of recombinant tau with different aggregation statuses exert persistent disruptive actions on synaptic plasticity, first, we examined the effect of SτAs on LTP induced by our standard HFS-200 Hz protocol in WT rats. A single injection of SτAs (19.6 pmol i.c.v.) had a persistent disruptive action 7 days later (103 ± 2.3% at 3 h post-HFS; compared with 134.3 ± 6.6% in the vehicle group (10 μl, i.c.v.) and with 143.3 ± 9.0% in mTau-injected animals), see figure 3*b,c*. We also assessed the ability of HFS-200 Hz to induce LTP in WT rats seven days after a single injection of the oTau preparation (oTau, 54 pmol, i.c.v.). Similarly, HFS triggered a decremental LTP one week after oTau injection (108.8 ± 3.4% at 3 h post-HFS; *p* = 0.0296, paired *t*-test; *n* = 5; data not shown).

In a longitudinal design, we examined if SτAs exerted the same disruptive action against LTP induced by the strong 400 Hz protocol in awake freely behaving WT and APP TG 5–10-month-old rats with chronic implants. In both WT and TG rats that received a single i.c.v. injection of 19.6 pmol of mTau, the repeated application of sHFS-400 Hz, both immediately (15 min) after the injection and two weeks later, triggered robust LTP similar in magnitude to that induced by the application of sHFS prior to the injection (electronic supplementary material, figure S2*a*). By contrast, LTP was inhibited both immediately and two weeks after the injection of the same dose of SτAs in both WT and TG rats (electronic supplementary material, figure S2*b*). As shown in the electronic supplementary material, figure S2*c*, in a follow-on study seven weeks after the injection of SτAs, sHFS-400 Hz induced stable LTP in a sub-cohort of these WT rats, whereas LTP was strongly blocked in a sub-cohort of the TG rats (139.9 ± 8.9% in WT versus 93.5 ± 20.9% in TG).

### (d) Sonicated tau aggregate-evoked impairment of novel object recognition and Y-maze performance

The possibility that the same SτA dose (19.6 pmol) would also cause disruption of cognition was tested sequentially over a few days. As illustrated in the study design shown in figure 3*d*, three behavioural tests were employed: open field (OF) exploration, novel object recognition (NOR) and Y-maze alternations. Performance in the OF test on day 0, 2  h after the i.c.v. injection was similar in mTau- and SτA-injected rats. Both groups travelled a similar distance (lines crossed, 113.8  ±  8.6 in mTau, versus 123.5 ± 10 in oTau; see figure 3*e*), had similar number of entries into the centre (7.1 ± 0.8 in mTau, *n* = 8 versus 8.8 ± 1.1 in oTau; *n* = 10; unpaired *t*-test, *p* = 0.2571; data not shown) and spent a comparable amount of time in the centre (25 ± 4.7  s in mTau, *n* = 8 versus 25.7 ± 2.9  s in oTau; *n* = 10; unpaired *t*-test, *p* = 0.8966; data not shown).

The next day, rats treated with mTau had a small but significant bias towards the novel object when tested 3  h after the training session (0.6756 ± 0.06 discrimination index (DI), figure 3*f*). By contrast, and consistent with the LTP studies, SτA-treated rats failed to have a significant preference for the novel object (0.4973 ± 0.07 DI).

Similarly, in the Y-maze on day 2, whereas mTau-injected rats entered the arms sequentially (72.6 ± 5.4% alternations), the rats that had been injected with SτAs did not (64.3 ± 6.3%; see figure 3*g*).

### (e) Antibody-induced rapid reversal of persistent inhibition of standard 200 Hz conditioning stimulation (HFS-200 Hz)-induced long-term potentiation by Pick’s disease-derived brain tau

**Figure 4.**
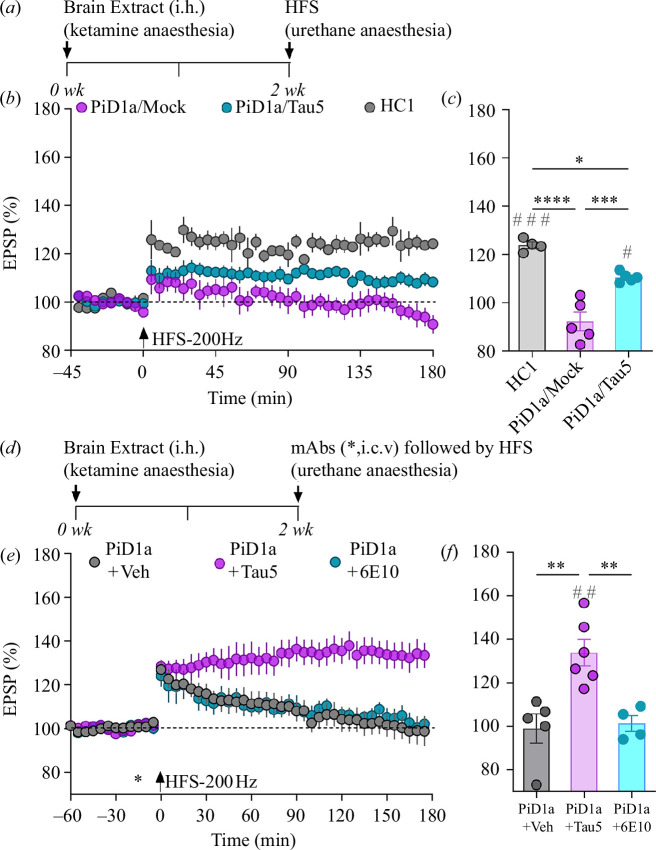
Persistent inhibition of HFS-200 Hz-induced LTP by PiD aqueous brain extract is tau-dependent and rapidly reversed by an anti-tau antibody. (*a*) Study design. WT animals received a single injection (i.h., 5.5 μl) of an extract from a healthy control brain (HC1), PiD1a extract immunodepleted of tau with Tau5 (PiD1a/Tau5) or PiD1a extract mock-immunodepleted with 46-4 (PiD1a/Mock) under recovery anaesthesia. Fourteen days later, the ability of our standard HFS (single train at 200 Hz, arrow) to induce LTP was assessed. Time course graphs (*b*) and summary bar charts (*c*). (*d*) Study design. All animals received mock-immunodepleted PiD1a extract (PiD1a, i.h., 5.5 μl) under recovery anaesthesia. Fourteen days later, animals received an injection of vehicle (Veh), Tau5 or an isotype control (6E10, an anti-Aß antibody) via the i.c.v. (*) route (2.5 μg of antibody) 15 min prior to HFS. (*e*,*f*) LTP disruption was rapidly reversed by injecting Tau5 but not 6E10. Values are mean ± s.e.m. ^#^ *p* < 0.05, ^##^ *p* < 0.01, ^###^ *p* < 0.001 compared with pre-HFS, paired *t*‐test; *p < 0.05, **p < 0.01, ***p < 0.001, ****p < 0.0001 one-way ANOVA followed by multiple-comparison tests.

Neuropathologically, AD is defined as a tauopathy on a background of ß-amyloidosis. Other neurodegenerative diseases present as relatively pure tauopathies, including PiD. Having previously reported that tau in an aqueous brain extract from a PiD patient (PiD1a) acutely inhibited LTP in an Aß-independent manner [14], we wondered if such tau persistently inhibited LTP, similar to what we reported above for recombinant tau. Two weeks after a single intrahippocampal (i.h.) injection of PiD1a extract that had been mock-ID of tau with an isotype control mAb (PiD1a/Mock), but not the same volume (5.5 µl) of an extract from a healthy control (HC1), the application of our standard HFS-200 Hz protocol in acutely anaesthetized rats failed to induce significant LTP (92 ± 3.8% versus 123.9 ± 1.3%; figure 4*b,c*). By contrast, partial tau ID of the extract with the mAb Tau5 (PiD1a/Tau5; see the electronic supplementary material, table S3) partly prevented the inhibition of LTP (109 ± 2.3% at 3 h post-HFS), confirming a critical role for tau in mediating the persistent disruption of synaptic plasticity.

To determine if the persistent inhibition of LTP was reversible using anti-tau antibody treatment, we applied an acute i.c.v. injection of Tau5 two weeks after the i.h. injection of PiD1a, just 15 min prior to HFS. Remarkably, robust LTP was now induced (137.2 ± 6.2%), whereas PiD1a extract still strongly inhibited LTP (101.3 ± 3.6%) in animals that received an acute i.c.v. injection of an isotype control anti-Aß mAb, 6E10 (figure 4*e,f*).

### (f) Antibody-induced rapid reversal of persistent inhibition of HFS-200 Hz-induced long-term potentiation by Alzheimer’s disease brain tau and amyloid ß protein

**Figure 5.**
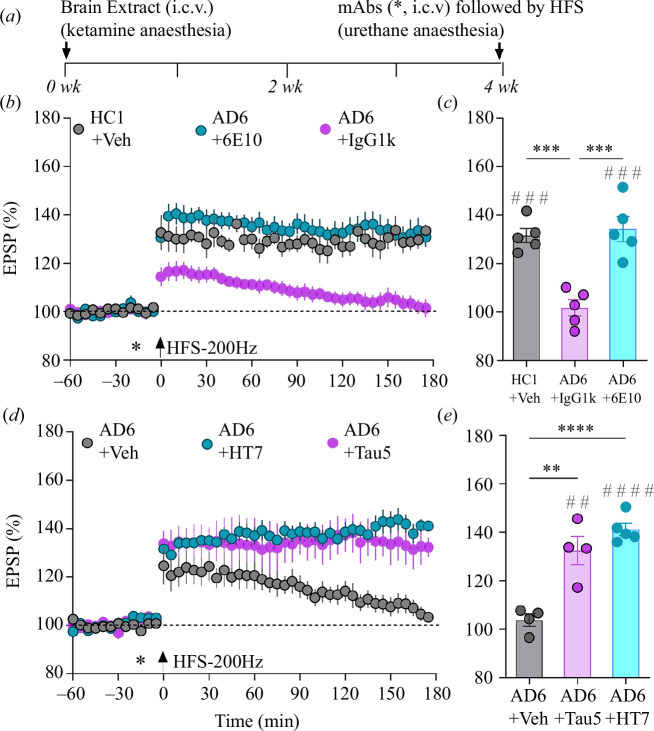
Synaptotoxic Aß- and tau-containing AD brain aqueous extract-mediated persistent inhibition of HFS-200 Hz-induced LTP is rapidly reversed by anti-Aß and anti-tau antibodies. (*a*) Study design. WT animals received an injection (i.c.v., 11 μl) of an extract from a healthy control (HC1) or an AD patient (AD6) under recovery anaesthesia. (*b*,*c*) Twenty-eight days later animals were injected with vehicle (HC1+Veh), an isotype control monoclonal antibody IgG1k (AD6+IgG1k) or 6E10 (AD6+6E10) via the i.c.v. (*) route (2.5 μg of antibody) 15 min prior to HFS (single train at 200 Hz, arrow). (*d*,*e*) The same protocol was followed for anti-tau antibodies Tau5 (AD6+Tau5) and HT7 (AD6+HT7) or vehicle (AD6+Veh). HFS-200 Hz triggered stable LTP in animals injected with either Tau5 or HT7 but not vehicles. Values are mean ± s.e.m. ^##^ *p* < 0.01, ^###^ *p* < 0.001, ^####^ *p* < 0.0001 compared with pre-HFS, paired -test; **p < 0.01, ***p < 0.001, ****p < 0.0001 one-way ANOVA followed by multiple-comparison tests.

Finally, we tested the reversibility of the persistent inhibition of LTP by AD6, (see above and the electronic supplementary material, tables S1 and S2), that acutely and persistently inhibits LTP in a manner that requires both Aß and tau [14]. As shown in figure 5*b,c*, we found that four weeks after a single i.c.v. injection of AD6 extract (11 µl), the application of HFS-200 Hz failed to induce stable LTP (101.8 ± 3.3%). By contrast, robust LTP (131.5 ± 2.9%) was induced in rats four weeks after a similar injection of extract from the healthy control (HC1; figure 5*b,c*). In the case of AD6, consistent with the presence of synaptotoxic Aß, unlike PiD1a, i.c.v. injection of 6E10, an anti-Aß mAb enabled the induction of LTP (134.3 ± 5.2%) by HFS when applied 15 min later (see figure 5*b,c*). Importantly, the anti-tau mAb Tau5 (i.c.v.) also rapidly reversed the deficit in LTP four weeks after the injection of the AD6 extract (141.2 ± 2.5% versus 104.1 ± 2%, respectively; figure 5*d,e*). Because Tau5 recognizes an epitope of tau that is shared between rats and humans (tau210-241), we also tested a different anti-tau mAb, HT7, that targets a human tau sequence (tau159-163) not shared with rats. Significantly, as shown in figure 5*d,e*, HT7 also reversed the persistent impairment of LTP caused by AD6 extract (132.5 ± 5.8%).

## 3. Discussion

The finding that relative to WT rats, oTau was a more potent disrupter of sHFS-400 Hz-induced LTP in APP TG animals at an age when oAß is elevated but prior to the formation of extensive fibrillar Aß deposits in plaques [25,28] may indicate that endogenous oAß lowers the threshold for oTau to acutely disrupt synaptic plasticity. In the case of SτAs, we tested a relatively large dose in a longitudinal study. Although the SτAs exerted similar acute inhibitory effects in both genotypes, the rate of spontaneous recovery from the disruptive action of SτAs appeared much slower in the TG rats. Thus, oTau and SτAs may have enhanced neuroactivity on a background of elevated endogenous oAß. However, when we assessed the disruptive effect of synaptotoxic tau in AD brain aqueous extracts in APP versus WT rats, we found variable results that depended on the sample. For AD1 extract, which inhibits LTP in a tau-dependent and Aß-independent manner (present findings and [11]), both genotypes were similarly affected. It would be instructive to test a lower volume of this extract that is subthreshold in WT rats to determine if the threshold is sensitive to genotype. On the other hand, for AD6 extract, which inhibits LTP in both a tau- and Aß-dependent manner ([14] and present findings), a relatively large volume of extract was required to inhibit sHFS-induced LTP in WT animals, and this threshold volume failed to exert a disruptive action in TG rats. Because this extract required the presence of both synaptotoxic tau and Aß to inhibit 400 Hz-sHFS-induced LTP in WT rats, a reduction in the activity of either protein would be sufficient to explain the loss of efficacy of AD6 extract in APP rats. A loss of responsiveness to tau seems less likely since synaptotoxic tau in the other sources (AD1 and oTau) maintained its activity in the TG rats. To determine if the presence of synaptotoxic oAß in AD6 extract was a significant factor in raising the threshold in TG rats, future experiments may wish to assess the ability of synthetic oAß to inhibit LTP in pre-plaque TG compared with WT rats. We know from others that when plaques become abundant, fibrillar Aß may act as a sink for more soluble forms of Aß [29,30], potentially neutralizing synaptotoxic oAß. In the present study, because Aß fibrils are unlikely to be abundant in the TG rats at the age tested here [25,31], the latter explanation may not explain the current findings.

Our discovery that a single intracerebral injection of SτAs, but not mTau, disrupted LTP for at least two weeks post-injection in WT rats may indicate that these tau aggregates persist in the brain for long periods. SτAs have been reported to act as seeds for the prion-like spread of tauopathy but such effects often require the presence of an amyloid background [32–34]. In contrast to seeding of neuropathological proteins, which usually takes many weeks to reveal itself, reaching a peak after some months (e.g. [19,33,35]), as seen here the onset of tau synaptotoxicity was virtually immediate, persisted for some weeks and appeared to wane, at least in the case of SτAs in WT rats, between one and two months after the injection. One interesting aspect of our discovery of the long-lasting disruption is that it enables the testing of potential therapeutic interventions to reverse the synaptotoxicity of tau for some weeks after tau application. Remarkably, the deficit in LTP was fully reversed by a single injection of anti-tau antibody two to four weeks after the administration of either a PiD or an AD brain aqueous extract. That one of the active anti-tau antibodies was human sequence selective strongly implicates the continued presence of the synaptotoxic tau in the AD extract AD6 that was injected four weeks previously. Future studies should verify this hypothesis directly, by sensitive Western blotting, for example. The presumed continued presence of synaptotoxic tau species in our rats would indicate that the clearance mechanisms for these tau aggregates are relatively inefficient. In the case of AD6 brain extract, which inhibits LTP in an Aß- as well as a tau-dependent manner, a human-selective antibody that recognizes Aß also rapidly reversed the deficit. This finding is consistent with our previous finding that a persistent inhibition of LTP caused by i.c.v. injection of synthetic oAß was rapidly reversed by agents that targeted putative downstream synaptotoxic mechanisms [28].

As shown here and previously [9–11,22], both oTau and SτAs mimic the LTP disruptive action of synaptotoxic tau in patient-derived brain extracts [9,11,22] and extracellular tau in secretomes of induced pluripotent stem cell-derived neurons from individuals with trisomy 21, the most common genetic cause of AD [36], but see [37]. Whether or not the responsible tau species are the same remains to be determined. Our present AFM findings, based on the sample preparation described, are consistent with previous characterization of oTau [9,38] and SτAs [11,22] in so far as the latter appear as larger particles, with some overlap in size. It is feasible that the shared ability of oTau and SτAs to inhibit LTP is caused by a shared species in the two preparations. Nevertheless, ongoing unpublished research in our laboratory indicates that oTau can mimic the long term depression (LTD) facilitating action of synaptotoxic tau in patient brain extracts, unlike SτAs [26]. It is also likely that different species of tau may play a predominant role in mediating synaptotoxicity in different stages or subtypes of neurodegenerative diseases with tauopathy [8,15,39,40]. A related important area of ongoing research is the determination of the extent to which synaptotoxic tau shares cellular actions with synaptotoxic Aß [22,41].

In conclusion, we have found that synaptotoxic tau from recombinant protein or in patient-derived aqueous extracts shares the ability to acutely and persistently inhibit LTP. Furthermore, the presence of oAß in early amyloidosis appears to lower the threshold for tau-mediated LTP impairment. The ability of acute injection of human-specific anti-tau and anti-Aß antibodies to rapidly reverse the persistent synaptic plasticity disruption caused by AD brain extract points to the continued presence of synaptotoxic tau and Aß species. It also underlines the critical role of these species in the maintenance of synaptic plasticity disruption and helps inform future options for the development of therapeutic interventions.

## 4. Material and methods

### (a) Animals, cannula and electrode implantation and sample injection procedure

All experiments were conducted in accordance with ARRIVE (Animal Research: Reporting of In Vivo Experiments) guidelines under the approval of Trinity College Dublin local animal research ethics committee and the Health Products Regulatory Authority in Ireland, using methods similar to those described previously [14,42]. Rats were group-housed unless otherwise stated. Food and water were available *ad libitum* with a 12 L : 12 D cycle. For most experiments, we used 1.5–7-month-old male Lister hooded (LH; 96 animals in total) and 5–7-month-old male McGill-R-Thy1-APP TG rats (*n* = 34) expressing human APP751 with Swedish and Indiana mutations under the control of the murine Thy1.2 promoter [25]. Female LH rats were studied in the behavioural experiments (see §4g). We previously reported that synaptic plasticity in LH rats is very sensitive to the disruptive action of disease-relevant tau species. We chose McGill-R-Thy1-APP rats to study the synaptotoxicity of exogenous tau in animals with a relatively high endogenous human Aß background. The TG animals were genotyped commercially by Transnetyx (Cordova, TN, USA) using real-time polymerase chain reaction. McGill-R-Thy1-APP rats have an age-dependent accumulation of Aß, first as oligomers and gradually as fibrils in neuritic plaques starting after six months of age in some homozygous animals [25,31]. LH and McGill WT (littermates of TG animals, *n* = 46) rats were combined to form a WT group for comparison with age-matched TG rats. In the studies examining the effect of amyloidosis on the response to tau in freely behaving animals, we studied older (5–10-month-old) TG (*n* = 9) and WT McGill male rats (*n* = 11). LH females (2–3-month-old, 24 animals in total) were studied in the experiments investigating the effects of tau on baseline synaptic transmission and behaviour.

To inject samples and test synaptic plasticity, the animals were anaesthetized with urethane (1.6 g kg^−1^, intraperitoneal (i.p.)), and core body temperature was maintained at 37.5 ± 0.5°C. An i.c.v. stainless steel guide cannula (22 gauge, 0.7 mm outer diameter, length 13 mm) was implanted above the right lateral ventricle (coordinates, 0.5 mm posterior to bregma and 1.2 mm right of midline, depth 4 mm) before the electrodes were implanted ipsilaterally. Teflon-coated tungsten wire (external diameter 75 µm bipolar or 112 μm monopolar) electrodes were positioned in the stratum radiatum of area CA1. The electrodes were optimally located using a combination of physiological and stereotactic indicators (3.8 mm posterior to bregma and 2.5 mm lateral to midline, 4.6 mm posterior to bregma and 3.8 mm lateral to midline for recording and stimulating electrodes, respectively). Screw electrodes located over the contralateral cortex were used as reference and earth. To inject samples acutely, a Hamilton syringe was connected to the internal cannula (28 gauge, 0.36 mm outer diameter). The injector was removed 1 min post-injection and a stainless-steel plug was inserted.

To investigate the persistence of the effects of human brain extracts and recombinant tau preparations, the i.c.v. (coordinates as above) or i.h. (two separate injections: 4.5 mm posterior to bregma and 3.5 mm right of midline, depth 3.0 mm; and 4 mm posterior to bregma and 3 mm right of midline, depth 2.5 mm) injections were conducted under recovery anaesthesia using a mixture of ketamine and medetomidine (60 and 0.4 mg kg^−1^, respectively, i.p.), and the cannula was then removed. Afterwards, rats were housed individually in their home cages.

The timing, route and doses/volumes of injection of antibodies, recombinant tau and patient-derived brain aqueous extracts were based on pilot experiments, our previous experience with these materials and models [11,14,26–28,42–45] and the available scientific literature [9,41,46].

### (b) Preparation of aqueous human brain extracts and immunodepletion

Human brain tissue was used in accordance with Trinity College Dublin, Faculty of Health Science Ethics Committee (under approval 16014) and the Harvard University Partners Institutional Review Board (protocol, Walsh BWH 2011) guidelines. Frozen tissue was obtained from four cases, two of whom died with end-stage AD (referred to as AD1, AD6), one with Pick’s disease (PiD1) and one individual who died free of any signs of neurodegeneration (healthy control 1 (HC1)). All diseased cases met current post-mortem diagnostic criteria for the relevant disorder (see table 1 in ref. [14]). Brain samples from AD1, PiD1 and HC1 were obtained from the Massachusetts Alzheimer’s Disease Research Center Neuropathology Core at Massachusetts General Hospital. AD6 brain was from the Banner Health brain bank. Aqueous brain extracts were prepared in Dr Dominic Walsh’s laboratory by cortical grey matter tissue homogenization, centrifugation and dialysis (2K molecular weight cut-off) as described in our previous publications [11,14,22].

Extracts of AD6 and PiD1 (renamed PiD1a here to distinguish it from a different extract from the same donor, PiD1, published previously, [22]) brains, in 0.5 ml aliquots, were depleted of tau by three rounds (8, 16 and 8 h) of incubations with the anti-tau mAb Tau5 (10 μg) and Protein G agarose (PAG; 10 μl; Roche) beads, as described in our previous publications [14,22]. In parallel, a portion of these extracts was mock immunodepletion using 10 μg of an isotype control mAb, 46-4 [47] and PAG (10 μl). The Tau5 and 46-4-treated samples were cleared of beads and then incubated with PAG alone to remove residual IgG. The aliquots were stored at −80°C until used for LTP experiments. For AD6 extract, we combined data for treatments with mock ID for Aß or tau, using pre-immune serum and 46-4, respectively.

The aqueous brain extracts were characterized in terms of tau content and efficiency of ID using the Tau (Total) ELISA Kit (catalogue no. KHB0041, Invitrogen), see the electronic supplementary material, table S3 and [14] for details and values.

### (c) Oligomer-enriched tau

Oligomers of recombinant WT full-length tau-441 (2N4R, MW = 45.9 kDa) were prepared following a previously published protocol [48]. Briefly, human recombinant tau (R&D, SP-495) solution with a starting concentration of 2 mg ml^−1^ was initially treated with the reducing agent TCEP (final concentration 1 mM, Thermo Scientific, 20490) and 5 mM EDTA (Millipore, 324506) at 37°C for 1 h. Following the reduction step, TCEP and EDTA were eliminated using a 10K MWCO protein concentrator (Thermo Scientific, 88513). The tau protein was oligomerized by oxidative cross-linking with 1 mM H_2_O_2_ at room temperature for 20 h. Next, we performed buffer exchange to remove excess chemicals in a refrigerated centrifuge (12 000*g*, 4°). Aliquots of tau solution were immediately frozen on dry ice and stored at −80°C.

### (d) Soluble sonicated tau aggregates

We investigated the effects of two different preparations of recombinant, full-length human tau: WT and P301S (P301S_103his-tag_avi-tag full-length tau441) tau, a more aggregation-prone sequence that is responsible for certain forms of familial dementia [49]. Both forms were expressed, purified and monomer concentrated in Dr Dominic Walsh’s laboratory as described previously [11]. Heparin was then added to promote aggregation and fibrils harvested by ultracentrifugation. Tau fibrils were ultrasonicated to prepare SτAs. In the majority of experiments, we only tested WT tau.

To quantify the monomeric tau equivalent present in aggregated tau preparations, 10 μl of each stock was diluted with 10 μl of 5 M guanidinium hydrochloride (final concentration of 2.5 M) and allowed to disperse overnight at 4°C. The following day, the concentration of tau in these recombinant preparations was measured using the Tau (Total) ELISA kit (Invitrogen, no. KHB0041), see the electronic supplementary material, table S3. Standards and samples were measured in duplicates.

### (e) Atomic force microscopy

For AFM measurements, samples of tau monomer, tau oligomers and sonicated tau aggregates were diluted in phosphate buffered saline (PBS) to a final concentration of 155, 162 and 177 µg ml^−1^, respectively. A 5 µl volume of each suspension was pipetted onto freshly cleaved mica (diameter 12 mm; Ted Pella, Redding, CA) surface and incubated at room temperature (nominally 21°C) for 2 min. Then the suspensions were blow-dried in a stream of nitrogen and followed by extensive rinsing with Milli-Q water. All imaging was performed using MFP-3D AFM (Asylum Research) in amplitude modulation mode at room temperature in ambient air with a Si AFM probe (NCH, Nanosensors) having a nominal resonance frequency of 320 kHz, and a spring constant of 42 N m^−1^. AFM height images were collected at randomly selected locations (3.5 × 3.5 µm, 256 × 256 pixels). AFM height images were flattened with a mask applied to the particles, using Igor software. Representative images are shown. No particles were found on freshly cleaved mica (not shown) after rinsing with Milli-Q water, which confirmed that the observed particles came from the solutions and not from the rinsing.

### (f) *In vivo* electrophysiology

#### (i) Non-recovery experiments

CA3-to-CA1 field excitatory postsynaptic potentials (EPSPs) were evoked and recorded in the stratum radiatum under urethane anaesthesia. LabChart 7 software controlled an analogue-to-digital system consisting of an in-house pre-amplifier (broadband range up to 4 kHz) connected to a PowerLab 2/26 (ADInstruments, Australia) for data acquisition and analysis. Single square-wave pulses (0.2 ms duration) generated by a constant current isolation unit PSIU6 (Grass Instruments Co., USA) were applied every 30 s at an intensity that triggered a 50% maximum EPSP response. To induce *N*-methyl-d-aspartate (NMDA) receptor-dependent LTP our standard 200 Hz conditioning stimulation protocol (HFS-200 Hz), consisting of one set of 10 trains of 20 pulses (inter-train interval of 2 s) at test intensity, was applied. A much stronger conditioning stimulation protocol (sHFS-400 Hz; three sets of 10 trains of 20 pulses at 400 Hz, the intertrain interval of 2 s, and the interset interval of 5 min and increased to 75% maximum intensity) was used to investigate LTP in APP transgenic rats and their WT littermates. This form of LTP is both NMDA-receptor-dependent and voltage-gated Ca^2+^-channel-dependent [45,50]. The magnitude of control LTP varied considerably over the period of carrying out these experiments. To minimize the possible confounding effect of such variation in control LTP, the experiments in each study were interleaved. There were no detectible abnormal changes in background hippocampal EEG which was monitored throughout the experiments.

To investigate the persistence of the effects of human brain extracts and recombinant tau, the injections of samples were conducted under recovery anaesthesia (see §4a). Subsequently, electrophysiological recordings were conducted under non-recovery urethane anaesthesia, as described above, 7 or 28 days later.

#### (ii) Longitudinal experiments

For the freely behaving recording in the longitudinal studies, the implantation procedure was carried out under recovery anaesthesia using a mixture of ketamine/medetomidine according to methods similar to those described previously [42]. The rats were allowed at least 14 days after surgery before recordings began. These rats were housed individually in their home cages post-surgery between recording sessions. Recordings were carried out in a well-lit room. The recording compartment consisted of the base of the home cage, including normal bedding and food/water, but the sides were replaced with a translucent Perspex plastic box (27 × 22 × 30 cm) with an open roof. The rats had access to food and water throughout the whole recording session from the same position as in the home cage. All animals were first habituated to the recording procedure over the post-surgery recovery period [42]. To induce LTP, we used the sHFS-400 Hz protocol, see §4f(i). In pilot studies, we confirmed our previous finding that repeated application of this protocol triggered similar magnitude LTP over several weeks in WT and TG rats [42].

### (g) Behavioural tasks

Three weeks before behavioural experiments female LH rats were implanted with a cannula in the lateral ventricle under recovery anaesthesia (co-ordinates and anaesthetic dose as above). After surgery, cannulated animals were individually housed. Each animal was handled for 5  min per day for a week prior to testing. For the OF, 2 h after the i.c.v. injection the rat was placed in the centre of an open square white apparatus (L × W × H, 60 × 60 × 40  cm) and allowed to explore it for 5  min. Three parameters were measured: number of lines (marked on the floor) crossed, number of entries into and time spent in the central area (35 × 35  cm). The next day, 24 h after the OF, the NOR test was performed in the same apparatus, using previously described methods [51]. During the sample phase, two identical objects were presented to the animal for 5  min. In the retention phase 3  h later, one of the familiar objects was replaced with a novel one and the animal was allowed to explore for a further 5  min. The DI was calculated as the duration of novel object interaction/duration of total interaction with both objects. Spatial working memory was assessed using a Y-maze 24 h after the NOR, 2 days after the i.c.v. injection. The Y-maze spontaneous alternation test employed methods described previously [52]. The symmetrical white Y-maze consisted of 3 arms positioned at a 120° angle from each other. Each arm was 58 cm long, 29 cm high, 16 cm wide. After an introduction to the centre of the maze, the animal was allowed to freely explore the three arms for 5 min. The number of arm entries and the number of triads were recorded in order to calculate the percentage of alternation as the number of triads/(number of entries − 2) × 100. An entry was counted when all four limbs of the animal were within the arm. A triad was counted when the animal entered all three arms consecutively. Between sessions, the maze was cleaned thoroughly with ethanol. In the behavioural tasks data from animals that failed to move for more than 15 s during the pre-defined 5 min test period were excluded from analysis.

### (h) Antibodies and chemicals

Tau5 (mouse IgG1, tau 210–241), 6E10 (mouse IgG1, human-Aß N terminal sequence in APP) and IgG1κ isotype control (clone MG1–45) antibodies were obtained from BioLegend. HT7 (mouse IgG1, human tau-specific residues 159–163) was purchased from Invitrogen. The isotype control mAb 46-4 (mouse IgG1, HIV–1 gp120) was obtained from Bio-Techne.

### (i) Data analysis

Values were presented as the mean ± s.e.m. percentage pre-injection (or pre-HFS in case of delayed-effects experiments) baseline EPSP amplitude over a 30–45 min period. The magnitude of LTP was measured at 3 h post-HFS and expressed as the mean ± s.e.m.% baseline. For graphing purposes, EPSP amplitude measurements were grouped into 5 min (average of 10 sweeps) or 10 min epochs (average of 20 sweeps in the case of freely behaving data in the electronic supplementary material, figure S1c. For statistical analysis, data are expressed as the average EPSP amplitude during the last 10 min epoch before and 170–180 min (3 h) after HFS. Sample sizes were chosen based on our previous publications with similar experimental designs [11,14]. The ability to induce LTP within each experimental group was assessed *a priori* using paired two-tailed *t*-tests. Differences in the magnitude of potentiation between experimental groups were analysed using one- or two-way ANOVA with Bonferroni’s *post hoc* tests or by unpaired two-tailed *t*-tests, as appropriate. One-sample *t-*tests were used to analyse the bias towards the novel object [53] or the ability to consecutively enter the arms of the Y-maze [52].

A *p*-value of <0.05 was considered statistically significant. Statistical analyses were performed in GraphPad Prism software (10.0.2).

## Data Availability

All data that are presented in the figures are referred to in the electronic supplementary material, table S1 [54].
